# Persistent hematuria in patients with antineutrophil cytoplasmic antibody-associated vasculitis during clinical remission: chronic glomerular lesion or low-grade active renal vasculitis?

**DOI:** 10.1186/s12882-017-0763-7

**Published:** 2017-12-06

**Authors:** Li Lv, Dong-Yuan Chang, Zhi-Ying Li, Min Chen, Zhao Hu, Ming-Hui Zhao

**Affiliations:** 1 Renal Division, Department of Medicine, Peking University First Hospital; Peking University Institute of Nephrology; Key Laboratory of Renal Disease, Ministry of Health of China; Key Laboratory of Chronic Kidney Disease Prevention and Treatment (Peking University), Ministry of Education, Beijing, 100034 China; 2 Qilu Hospital of Shandong University, Jinan, 250012 China

**Keywords:** ANCA, Vasculitis, Hematuria

## Abstract

**Background:**

Whether persistent hematuria in patients with antineutrophil cytoplasmic antibody (ANCA)-associated vasculitis (AAV) during clinical remission reflects active disease or chronic glomerular injury is uncertain. This study aimed to investigate the significance of persistent hematuria during clinical remission in a large cohort of AAV patients.

**Methods:**

A cohort of 219 AAV patients in complete clinical remission after induction therapy at our center was retrospectively studied, and their clinical and laboratory data as well as long-term outcomes were analyzed.

**Results:**

A total of 80 out of 219 patients had persistent hematuria during clinical remission of AAV. Compared with patients without hematuria during remission, the slope of eGFR decline in patients with persistent hematuria was significantly higher during the long-term follow-up [3.6 (IQR 1.2, 7.2) vs. 1.5 (IQR 0.2, 4.0) mL/min/1.73 m^2^/year, *P* < 0.001]. Among the 80 patients with persistent hematuria during remission, there was little difference between those with fast and slow decline of eGFR, as divided by either median or interquartile range of the slope of eGFR decline. We also compared patients without hematuria who had a slope of eGFR decline that was lower than the median level of the slope of eGFR decline with those with persistent hematuria, and found that patients with hematuria had significantly lower levels of CRP and ESR at baseline and higher levels of ANCA at remission.

**Conclusions:**

Among the AAV patients who achieved clinical remission after immunosuppressive therapy, those with persistent hematuria are not rare and may reflect either chronic renal damage or low-grade active renal disease.

## Background

Antineutrophil cytoplasmic antibody (ANCA)-associated vasculitis (AAV) is a group of systemic diseases characterized by small vessel inflammation and necrosis in combination with ANCA directed against proteinase 3 (PR3) or myeloperoxidase (MPO) [1]. AAV can affect many organs, especially the kidneys and lungs [2]. Patients with untreated AAV have a poor prognosis; the estimated average survival from onset of the disease was reported as 5 months, and the 2-year mortality rate reached approximately 90% [3]. Although the outcome of AAV has markedly improved with the introduction of corticosteroids and cyclophosphamide, over 20% of patients with AAV developed end-stage renal disease (ESRD) [4].

Renal involvement in AAV often manifests as hematuria, with or without elevation in serum creatinine. In clinical practice, remission of AAV was defined as improvement or stabilization in serum creatinine and resolution of hematuria [5]. However, when the active disease was controlled and corticosteroids tapered gradually, some patients still had microscopic hematuria. Currently, the significance of persistent hematuria in clinical remission of AAV still remains controversial, i.e., whether it reflects low-grade active renal disease or chronic glomerular injury is unknown [4, 6–9]. Geetha et al. analyzed 9 AAV patients with persistent hematuria during clinical remission who underwent re-biopsies for persistent hematuria. They found that none of these patients had active vasculitis, and persistent hematuria in remission of AAV patients may represent chronic glomerular injury [6]. Magrey et al. analyzed 10 of 25 patients with AAV who had sustained hematuria for more than 6 months despite apparent clinical remission. They found that persistent hematuria could be attributed to chronic glomerular damage in view of circumstantial evidence [8]. In another study of 55 AAV patients, Chen et al. [9] reported that hematuria duration during clinical remission was not predictive of estimated glomerular filtration rate (eGFR) at 1 year and concluded that persistent hematuria in remission could be a marker of chronic glomerular damage. Because of the relatively small sample sizes of these studies, to further elucidate this issue, we retrospectively analyzed the long-term outcomes of 219 AAV patients in clinical remission at our center.

## Methods

### Patients

Two hundred and nineteen consecutive patients with newly diagnosed AAV at the Renal Division, Peking University First Hospital between June 1996 and September 2015, were retrospectively included in the study. Inclusion criteria were as follows: (1) All patients met the Chapel Hill Consensus Conference nomenclature criteria for AAV [including granulomatosis with polyangiitis (GPA), microscopic polyangiitis (MPA), and renal-limited vasculitis (RLV)] [1]; (2) ANCA positivity; (3) clinical remission was achieved after induction therapy. A total of 97 patients underwent renal biopsy at diagnosis. The follow-up time of the cohort was from December 2002 to March 2016. Exclusion criteria were as follows [10]: (1) patients with eosinophilic granulomatosis with polyangiitis (EGPA) because EGPA is considered a distinct type of AAV with different manifestations and outcomes compared to GPA, MPA, and RLV [11]; (2) patients with secondary vasculitis such as propylthiouracil-induced AAV, or in combination with other renal diseases such as anti-glomerular basement membrane disease, IgA nephropathy, lupus nephritis, diabetic nephropathy, or membranous glomerulopathy. ANCAs were tested by both indirect immunofluorescence assay (IIF) and antigen-specific enzyme-linked immunosorbent assay (ELISA).

Treatment protocols have been previously described [12, 13]. In brief, induction therapy included corticosteroids and cyclophosphamide (CTX). Patients with severe pulmonary hemorrhage or acute renal failure requiring dialysis at diagnosis received additionally plasma exchanges. For maintenance therapy, intravenous CTX every three months, or daily oral azathioprine (AZA) or mycophenolate mofetil (MMF) was administered.

### Definitions of disease activity

Hematuria was defined based upon greater than 3 dysmorphic red blood cells in urine per high power field under a microscope, which was measured every 1–3 months. The microscopic examination of the urine was performed by trained nephrologists. If the microscopic examination of urine showed the red blood cells were mainly monomorphic, the patients would receive ultrasonic examination or even endoscopy, and thus urologic causes of hematuria were excluded. Renal remission in vasculitis in this setting included stabilization or decrease of the serum creatinine concentration, concurrent with gradual tapering of immunosuppressive medications, even if the patient experienced continued proteinuria or microscopic hematuria, as previously described [8]. Relapse was defined as the recurrence or new onset of disease activity attributable to active vasculitis, or worsening of disease activity [14]. ESRD was defined as a continuous (greater than 3 months) need for renal replacement therapy. eGFR was calculated as previously described [15]. Disease activity at the time of diagnosis was scored according to the Birmingham Vasculitis Activity Score (BVAS) [16]. The slope of eGFR decline was defined as eGFR at the beginning of maintenance therapy minus eGFR at last follow-up divided by years. The end-point of renal survival was defined as a 100% increase in serum creatinine or >50% decrease in eGFR compared with that in remission or ESRD.

Renal biopsies were performed before the initiation of immunosuppressive therapy. Renal histology of AAV patients was evaluated according to the previously standardized protocol [17–19]. In short, each glomerulus was scored separately on the presence of fibrinoid necrosis, crescents, glomerulosclerosis, granulomatous reactions, as well as a number of other lesions. The presence of glomerular lesions was calculated as the percentage of the total number of glomeruli in a biopsy. Interstitial and tubular lesions were scored semi-quantitatively according to the percentage of the tubulointerstitial compartment that was affected: interstitial infiltrates (‘-’ for 0%, ‘+’ for 0–20%, ‘++’ for 20–50% and ‘+++’ for >50%), interstitial fibrosis (‘-’ for 0%, ‘+’ for 0–50%, ‘++’ for >50%) and tubular atrophy(‘-’ for 0%, ‘+’ for 0–50%, ‘++’ for >50%).

### Statistical analysis

Values are expressed as the mean ± standard deviation (SD) and median [interquartile range (IQR)] for continuous normally and non-normally distributed variables, respectively. Differences in quantitative parameters between groups were assessed with the t-test (for normally distributed data) or non-parametric test (for non-normally distributed data). Differences in qualitative results were compared with the chi-square test. Kaplan-Meier survival analysis was performed to analyze the renal outcome. Multivariate analysis of predictors of the renal outcome was performed with Cox proportional hazards regression and results are expressed as hazard ratios (HRs) with 95% confidence intervals (CI). *P* values less than 0.05 were considered statistically significant. Statistical analysis was performed using SPSS statistical software package (version 13.0, Chicago, IL).

## Results

### General data

Of the 219 patients with AAV, 89 (40.6%) were male and 130 (59.4%) were female, with an age of 58.5 ± 13.7 (range 18–86) years at diagnosis. One hundred and ninety-five of the 219 (89.0%) patients were positive for pANCA, and all of those sera recognized MPO in an antigen-specific ELISA. Twenty-four of the 219 (11.0%) patients were positive for cANCA, and all of those sera recognized PR3 in an antigen-specific ELISA. The median duration of follow-up was 37.0 (IQR 22.0, 57.8) months. All of these patients achieved complete remission of extra-renal vasculitis and stabilization or decrease of the serum creatinine concentration after the standard induction therapy, concurrent with gradual tapering of immunosuppressive medications. A total of 128 out of 219 patients achieved hematuria resolution; 80 patients had persistent hematuria; and 11 patients had new-onset hematuria during the remission stage. Among the 11 patients with new-onset hematuria, 4 patients were diagnosed as having renal relapse according to their clinical and laboratory data, and one patient was diagnosed with IgA nephropathy by re-biopsy.

### Comparison of patients with persistent hematuria and without hematuria during clinical remission of AAV

Because the sample size of patients with new-onset hematuria during clinical remission was small (*n* = 11), we did not include them in the following comparison.

We first compared the baseline data of patients with persistent hematuria and without hematuria during clinical remission of AAV. Compared with patients without hematuria, those with persistent hematuria had a significantly lower prevalence of weight loss and nervous system involvement, significantly lower levels of leukocytes and neutrophils in complete blood count, and significantly more severe interstitial fibrosis (Table 1 and Table 2). We also compared data at the beginning of the maintenance therapy between these two groups of patients. We found that patients with persistent hematuria had a significantly higher level of ANCA than patients without hematuria [expressed as median and IQR, 42.5 (0.0, 81.8) vs. 0.0 (0.0, 51.0) RU/mL (normal range 0–20 RU/mL, *P* = 0.017] (Table 1). However, such levels of ANCA were fairly low because the normal range of the ANCA test is below 20 RU/mL. The ANCA levels were significantly lower at the beginning of the maintenance therapy than at diagnosis [42.5 (IQR 0.0, 81.8) vs. 187.0 (IQR 96.0, 200.0) RU/mL, *P* < 0.001].

**Table 1:** General data of AAV patients

Parameters	With persistent hematuria in remission (*n* = 80)	Without hematuria in remission (*n* = 128)	*P* value
Baseline data			
Sex (Male/Female)	31/49	51/77	0.88
Age (years)	57.2 ± 12.3	59.4 ± 14.5	0.27
Hypertension (n %)	20/25.0	45/35.2	0.12
Cardiovascular disease (n %)	11/13.8	19/14.8	0.83
Diabetes (n %)	10/12.5	26/20.3	0.15
Using ACEI/ARB (n %)	18/22.5	22/17.2	0.34
ANCA subtypes (PR3/MPO)	11/69	13/115	0.43
ANCA level (RU/mL)	187.0 (96.0,200.0)	143.5(88.8,200.0)	0.37
BVAS	19.1 ± 6.9	18.4 ± 6.8	0.58
Hemoglobin (g/L)	97.5 ± 21.2	101.4 ± 20.5	0.22
Leukocyte (×10^9^/L)	8.5 ± 2.9	9.7 ± 3.2	**0.011**
Neutrophil (×10^9^/L)	6.4 ± 2.9	7.4 ± 3.1	**0.04**
Platelets (×10^9^/L)	259.4 ± 102.3	267.9 ± 98.3	0.59
ESR (mm/1 h)	56.5(43.5,79.8)	80.0(35.0,104.0)	0.09
C-reactive protein (mg/L)	9.8(3.5,39.2)	16.3(7.2,64.2)	0.11
Urine protein excretion (g/24 h)	1.0(0.3,1.8)	0.9(0.6,2.1)	0.56
Serum creatinine (μmol/L)	167.5(111.5,341. 0)	154.5(95.1,312.0)	0.50
eGFR (mL/min/1.73 m2)^a^	39.2 ± 28.9	42.9 ± 31.1	0.39
Fever (n/%)	31/38.8	60/46.9	0.25
Fatigue (n/%)	36/45.0	74/57.8	0.07
Rash (n/%)	10/12.5	20/15.6	0.53
Weight loss (n/%)	31/38.8	69/53.9	**0.033**
Muscle pain (n/%)	13/16.3	31/24.2	0.17
Arthralgia (n/%)	15/18.8	25/19.5	0.89
Lung (n/%)	49/61.3	74/57.8	0.62
ENT (n/%)	37/46.3	59/46.1	0.98
Gastrointestinal tract (n/%)	23/28.8	39/30.5	0.79
Nervous system (n/%)	7/8.8	24/18.8	**0.049**
Prednisone-equivalent dose (g)	8.9 ± 4.0	9.2 ± 3.1	0.53
Oral CTX/IV CTX	8/72	19/109	0.07
Data at the beginning of maintenance therapy			
Serum creatinine (μmol/L)	115.5(96.5,145.0)	114.0(90.0,154.8)	0.68
eGFR (mL/min/1.73m^2^)	54.1 ± 21.2	55.8 ± 24.7	0.61
ANCA level (RU/mL)	42.5(0.0,81.8)	0.0(0.0,51.0)	**0.017**
ESR (mm/1 h)	20.0(11.0,33.0)	19.0(14.0,33.0)	0.65
C-reactive protein (mg/L)	3.0(1.0,8.0)	3.0(1.0,8.0)	0.86
Proteinuria (n/%)^b^	48(61.5%)	52(40.6%)	**0.004**
IV CTX every 3 mo/AZA/MMF	25/53/2	34/86/8	0.61
The slope of eGFR decline (mL/min/1.73 m^2^/y)	3.6(1.2,7.2)	1.5(0.2,4.0)	**<0.001**

*Abbreviations: AZA* Azathioprine, *CTX* Cyclophosphamide, *ENT* Ear nose or throat, *ESR* Erythrocyte sedimentation rate, *IV* Intravenous, *MPO* Myeloperoxidase, *MMF* mycophenolate mofetil; PR3: proteinase 3

The bold represents *P* value < 0.05

^a^eGFR (mL/min/1.73 m^2^) = 175 × (plasma creatinine)^-1.234^ × age ^−0.179^ × 0.79 (if female); ^b^Proteinuria defined as ≥1 + on dipstick testing

**Table 2:** Renal histology of AAV patients

Parameters	With persistent hematuria in remission (*n* = 45)	Without hematuria in remission (*n* = 52)	*P* value
Glomeruli (number)	28.0(20.5,38.5)	24.0(19.0,39.0)	0.36
Normal glomeruli (%)	30.4(22.7,60.4)	41.1(19.3,66.2)	0.88
Glomerular fibrinoid necrosis (%)	0.0(0.0,3.6)	0.7(0.0,8.2)	0.11
Cellular crescents (%)	41.7(16.9,59.3)	40.3(11.1,54.3)	0.83
Fibrous crescents (%)	0.0(0.0,5.6)	0.0(0.0,5,5)	0.92
Glomerulosclerosis (%)	2.3(0.0,13.7)	4.2(0.0,15.9)	0.86
Interstitial infiltrates (−/+/++/+++)	0/16/27/2	1/20/30/1	0.18
Interstitial fibrosis (−/+/++)	0/15/30	5/21/26	**0.012**
Tubular atrophy (−/+/++)	2/25/18	5/22/25	0.07

The bold represents *P* value < 0.05

**Fig. 1:**
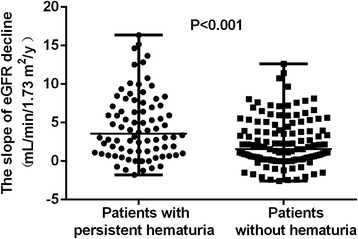
The slope of eGFR decline in AAV patients with persistent hematuria and without hematuria

**Fig. 2:**
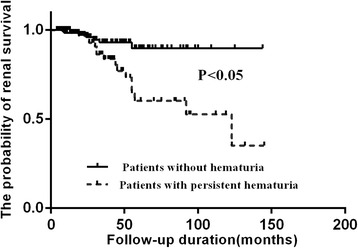
Renal survival in AAV patients with persistent hematuria and without hematuria

We then analyzed the outcomes of these two groups of patients. There was no significant difference in the initial eGFR or eGFR at the beginning of maintenance therapy between the two groups of patients. However, the slope of eGFR decline was significantly higher in patients with persistent hematuria than that in patients without hematuria [expressed as median and IQR, 3.6 (1.2, 7.2) vs. 1.5 (0.2, 4.0) mL/min/1.73 m^2^/year, *P* < 0.001] (Fig. 1). Accordingly, the Kaplan-Meier analysis showed that the renal survival was significantly worse in patients with persistent hematuria than in patients without hematuria (*P* = 0.003) (Fig. 2). After adjustment for age, sex, the previous history (cardiovascular diseases, hypertension and diabetes), using angiotensin-converting enzyme inhibitors (ACEI)/angiotensin II receptor blocker (ARB), proteinuria, and eGFR at baseline, persistent hematuria remained an independent predictor of the renal outcomes in the multivariate analysis (Table 3).

**Table 3:** Predictors of end-point events as analyzed by multivariate Cox regression

Factor	HR (95% CI)	*P* value
Age^a^	1.31(0.87,1.96)	0.19
Sex (male versus female)	1.25(0.45,3.46)	0.67
Hypertension (Yes/No)	1.40(0.50,3.91)	0.52
Cardiovascular disease (Yes/No)	1.81(0.56,5.93)	0.33
Diabetes (Yes/No)	0.38(0.07,2.00)	0.25
Using ACEI/ARB (Yes/No)	0.89(0.31,2.57)	0.83
Proteinuria (g/24 h)		
< 0.5	1.00 (ref)	Ref
0.5–1.0	0.49(0.09,2.81)	0.43
> 1.0	1.56(0.51,4.73)	0.44
Baseline eGFR (mL/min/1.73m^2^)^b^	0.73(0.59,0.91)	**0.005**
Persistent hematuria (Yes/No)	3.31(1.13,9.67)	**0.028**

*Abbreviations: ACEI* Angiotensin-converting enzyme inhibitors, *ARB* Angiotensin II receptor blocker, *eGFR* Estimated glomerular filtration rate

The bold represents *P* value < 0.05

^a^For every 10 years increase. ^b^For every increase by 10 mL/min/1.73 m^2^

### Further analysis of patients with persistent hematuria in clinical remission

In the present study, the slope of eGFR decline was relatively lower in 23 out of 80 patients with persistent hematuria who had a slope of eGFR decline <=1.5 mL/min/1.73 m^2^/year, which was the median slope of eGFR decline in patients without hematuria. The renal outcome in patients with persistent hematuria during clinical remission was heterogeneous, so we further analyzed the characteristics of this group of patients to identify some clues suggesting chronic glomerular injury or low-grade active renal vasculitis.

Among these 80 patients with persistent hematuria during clinical remission, two subgroups were identified according to the median slope of eGFR decline. We found little difference in baseline data between these two subgroups of patients, except for those with a higher slope of eGFR decline who had a higher prevalence of pulmonary involvement (72.5% vs. 50.0%, *P* = 0.039) and higher levels of urine protein excretion than those with a lower slope of eGFR decline [1.3 (IQR 0.7, 3.1) vs. 0.6 (IQR 0.3, 1.3) g/24 h, *P* = 0.001].

These 80 patients were then stratified according to quartile (Q1 through Q4) of the slope of eGFR decline. We compared the baseline data of patients in the first quartile with those of patients in the fourth quartile and found little difference between these two subgroups of patients, except that the levels of urine protein excretion were significantly higher in patients in the fourth quartile than those in the first quartile [1.1 (IQR 0.6, 2.8) vs. 0.3 (IQR 0.3, 1.0) g/24 h, *P* = 0.037].

Among the 128 patients without hematuria during remission, the median slope of eGFR decline was 1.5 mL/min/1.73 m^2^/year; 64 out of these 128 patients had a slope of eGFR decline <=1.5 mL/min/1.73 m^2^/year, and these patients can be reasonably considered as achieving the most complete renal remission and the slowest decline of renal function. We compared these 64 patients with the 80 patients with persistent hematuria during clinical remission. The slope of eGFR decline in these two groups of patients was 3.6 (1.2, 7.2) and 0.2 (−0.2, 0.8) mL/min/1.73 m^2^/year, respectively (expressed as median and IQR, *P* = 0.000). For the baseline data, patients with persistent hematuria had significantly lower levels of C reactive protein and erythrocyte sedimentation rates (ESR) than those without hematuria [9.8 (IQR 3.5, 39.2) vs. 18.4 (IQR 8.7, 62.6) mg/L, *P* = 0.043; 56.5 (IQR 43.5, 79.8) vs. 85.0 (IQR 45.5, 110.0) mm/1 h, *P* = 0.009, respectively). At the beginning of maintenance therapy, patients with persistent hematuria had a significantly higher level of ANCA than patients without hematuria [expressed as median and IQR, 42.5 (0.0, 81.8) vs. 0.0 (0.0, 37.4) RU/mL, *P* = 0.003] (Table 4).

**Table 4:** Comparison of patients with persistent hematuria and patients without hematuria

Parameters	With persistent hematuria in remission (*n* = 80)	Without hematuria in remission (*n* = 64)^a^	*P* value
Baseline data			
Sex (male/female)	31/49	27/37	0.68
Age (years)	57.2 ± 12.3	58.8 ± 14.5	0.50
Hypertension (n %)	20/25.0	23/35.9	0.15
Cardiovascular disease (n %)	11/13.8	8/12.5	0.83
Diabetes (n %)	10/12.5	13/20.3	0.20
Using ACEI/ARB (n %)	18/22.5	11/17.2	0.43
ANCA subtypes (PR3/MPO)	11/69	4/60	0.14
ANCA level (RU/mL)	187.0(96.0,200.0)	142.0(88.0,200.0)	0.14
BVAS	19.1 ± 6.9	19.0 ± 7.0	0.96
Hemoglobin (g/L)	97.5 ± 21.2	98.7 ± 19.6	0.76
Leukocyte (×10^9^/L)	8.5 ± 2.9	9.2 ± 2.7	0.19
Neutrophil (×10^9^/L)	6.4 ± 2.9	6.8 ± 2.5	0.41
Platelets (×10^9^/L)	259.4 ± 102.3	271.8 ± 108.0	0.53
ESR (mm/1 h)	56.5(43.5,79.8)	85.0(45.5,110.0)	**0.009**
C-reactive protein (mg/L)	9.8(3.5,39.2)	18.4(8.7,62.6)	**0.043**
Urine protein excretion (g/24 h)	1.0(0.3,1.8)	0.9(0.6,1.7)	0.62
Serum creatinine (μmol/L)	167.5 (111.5,341.0)	163.0(110.1,329.8)	0.10
eGFR (mL/min/1.73m^2^)	39.2 ± 28.9	39.2 ± 29.1	0.10
Fever (n/%)	31/38.8	23/35.9	0.73
Fatigue (n/%)	36/45.0	34/53.1	0.33
Rash (n/%)	10/12.5	9/14.1	0.64
Weight loss (n/%)	31/38.8	33/51.6	0.12
Muscle pain (n/%)	13/16.3	15/23.4	0.28
Arthralgia (n/%)	15/18.8	9/14.1	0.78
Lung (n/%)	49/61.3	35/54.7	0.47
ENT (n/%)	37/46.3	29/45.3	0.91
Gastrointestinal tract (n/%)	23/28.8	15/23.4	0.47
Nervous system (n/%)	7/8.8	11/17.2	0.13
Prednisone-equivalent dose (g)	8.9 ± 4.0	9.7 ± 3.2	0.23
Oral CTX/IV CTX	8/72	7/57	0.65
Data at the beginning of maintenance therapy			
Serum creatinine (μmol/L)	115.5(96.5,145.0)	122.0(95.3,160.3)	0.43
eGFR (mL/min/1.73m^2^)	54.1 ± 21.16	51.1 ± 22.5	0.42
ANCA level (RU/mL)	42.5(0.0,81.8)	0.0(0.0,37.4)	**0.003**
ESR (mm/1 h)	20.0(11.0,33.0)	19.0(15.0,34.0)	0.51
C-reactive protein (mg/L)	3.0(1.0,8.0)	3.0(1.0,5.8)	0.52
Proteinuria (n/%)^b^	48(61.5%)	22(34.4%)	**0.001**
IV CTX every 3 mo/AZA/MMF	25/53/2	17/42/5	0.45
The slope of eGFR decline (mL/min/1.73 m^2^/y)	3.6(1.2,7.2)	0.2(−0.2,0.8)	**<0.001**

*Abbreviations: AZA* Azathioprine, *CTX* Cyclophosphamide, *ENT* ear nose or throat, *IV* Intravenous, *MMF* Mycophenolate mofetil

The bold represents *P* value < 0.05

^a^This subgroup of patients included those with a slope of eGFR decline ≤1.5 mL/min/1.73 m^2^/y, which was the median slope of eGFR decline in AAV patients without hematuria. ^b^ Proteinuria defined as ≥1 + on dipstick testing

### Renal re-biopsy data

Three patients with persistent hematuria during clinical remission in our study received a renal re-biopsy because of suspected disease flare-up. All of them were treated with AZA in the maintenance therapy. According to findings at the renal re-biopsy, patient 1 was considered to have low-grade active renal vasculitis and was thus treated with an increased dose of prednisone in combination with AZA; the other 2 patients were considered to have chronic glomerular injury, and thus, the treatment remained unchanged (Table 5).

**Table 5:** Data of patients receiving renal re-biopsy

Patient (No.)	Age/gender	ANCA types	Organs involved	Initial Scr (μmol/L)	Duration of induction therapy (months)	Scr at the beginning of maintenance therapy (μmol/L)	Scr at re-biopsy (μmol/L)	Time interval of the re-biopsy (months)	Renal biopsy findings	Scr at last follow-up (μmol/L)
1	51/F	MPO	L,K,J	638	8	134	157	34	three cellular crescents, 10 cellular-fibrous crescents, 14 fibrous crescents in a total of 32 glomeruli	131
2	21/F	MPO	S,K	146	6	128	130	33	one cellular crescent, 1 cellular-fibrous crescent, 3 fibrous crescents in a total of 11 glomeruli	152
3	40/F	MPO	K	77	6	98	102	85	Three fibrous crescents in a total of 13 glomeruli	104

*Abbreviations L* Lung, *K* Kidney, *S* Skin, *J* Joint

## Discussion

Whether persistent microscopic hematuria in patients with AAV during clinical remission is a marker of low-grade active renal vasculitis or just a result of chronic glomerular injury has been controversial. This will, to a considerable extent, affect the treatment decision of whether to continue the aggressive immunosuppressive therapy, which is inevitably associated with an increased risk of infection and malignancy. The previous studies reported that persistent microscopic hematuria during remission can be a marker of chronic glomerular damage rather than active disease [6, 8, 9], and the authors proposed that patients who have achieved enduring remission despite persistent hematuria were allowed to taper or even withdraw their dose of medications. To further elucidate the significance of persistent hematuria during clinical remission of AAV, we retrospectively analyzed 219 AAV patients with clinical remission at our center and compared patients with persistent hematuria with those without hematuria.

In the present study, 80 out of 219 (36.5%) patients had persistent hematuria during clinical remission of AAV. This proportion was comparable to the previous study by Magrey et al. [8]. After the standard induction therapy, all of these patients had resolution of the extra-renal involvement of AAV and improvement *or* stabilization of serum creatinine, indicating that these patients achieved clinical remission, despite some of them having persistent hematuria.

It could be seen that the long-term renal outcomes of AAV patients with persistent hematuria during clinical remission were heterogeneous. Twenty-three out of 80 patients with persistent hematuria had a slope of eGFR decline <=1.5 mL/min/1.73 m^2^/year, which was the median slope of eGFR decline in patients without hematuria in the current study. This subgroup of patients had very stable renal function in the long-term follow-up, and their slope of eGFR decline was comparable to those without hematuria during remission. In this setting, persistent hematuria during remission was probably a sign of chronic glomerular damage, rather than active renal disease. This was consistent with the previous reports [6, 8, 9]. For these patients, it was advised that clinicians should gradually taper or withdraw corticosteroids and immunosuppressants [6, 8]. On the other hand, in some other patients with persistent hematuria, although clinical remission was achieved after aggressive immunosuppressive therapy, the renal outcome was still worse than those without hematuria. Indeed, the overall renal outcome in the long run was significantly worse in patients with persistent hematuria during clinical remission than those without hematuria, despite some of the abovementioned patients in the former group having a fairly good renal outcome. In the current study, we found that during remission, patients with persistent hematuria had higher levels of ANCA than those without hematuria. Therefore, the possibility that some of the patients with persistent hematuria still have low-grade active nephritis could not be fully excluded, although apparent remission has been achieved clinically. Whether prolonged aggressive immunosuppressive therapy should be employed in these patients remains to be determined.

Because the long-term renal outcomes of AAV patients with persistent hematuria during clinical remission were heterogeneous, it is clinically pertinent to differentiate the two clinical phenotypes among these patients, i.e., with very stable renal function or relatively fast decline of eGFR, which may represent chronic glomerular injury or low-grade active renal vasculitis, respectively. In the current study, among the patients who received renal re-biopsy, both of these circumstances existed. However, we found little difference between these two subgroups of patients, either in the baseline data or data in remission. Therefore, it is a therapeutic dilemma for these patients whether to initiate prolonged aggressive immunosuppressive therapy. In this circumstance where the status of renal disease activity was uncertain, renal biopsy is needed for those suspected of active nephritis.

In the study, we found that persistent hematuria in clinical remission was associated with the worse long-term renal outcomes. However, we found little difference between these two groups of patients in the baseline data, except that patients with persistent hematuria have lower levels of C reactive protein, ESR and leukocytes. We speculated that the reason might be active and fresh disease has better response to immunosuppressive therapy.

Regarding the role of proteinuria in AAV, in a number of previous studies, renal remission was defined as a stabilization or improvement of creatinine clearance in combination with the absence of erythrocyte cell casts [5, 6, 8, 20–22], and proteinuria was usually not included in a definition of renal remission. But this viewpoint remains much controversial. In the current study, patients with persistent hematuria in clinical remission have significantly higher proportion of proteinuria than those without hematuria. Without the data of re-biopsy in the majority of these patients, it is difficult to exclude that some of these patients, despite achieving clinical remission, had low-grade active renal vasculitis. Though proteinuria is a major factor in the progression of kidney damage, and inhibition of the renin-angiotensin-aldosterone system may improve the renal outcome [23–25], we did not found that the use of ACEI/ARB or proteinuria was independently associated with the long-term renal outcomes of AAV patients in the study. It is possibly due to the relatively short duration of follow-up, or the rapidly progressive nature of renal function of ANCA-associated glomerulonephritis which goes beyond the role of proteinuria.

There are several limitations for the present study. First, it is a retrospective study, which might result in the information bias. Second, as mentioned above, since the majority of patients did not receive renal re-biopsy, we did not have the “golden standard” to evaluate the activity of renal disease, especially for those with proteinuria.

## Conclusions

Among the AAV patients who achieved clinical remission after standard immunosuppressive therapy, those with persistent hematuria are not rare. This persistent hematuria may reflect either chronic renal damage or low-grade active renal disease, and the differential diagnosis of these two circumstances is sometimes difficult without renal histology.

## Abbreviations

AAV: Antineutrophil cytoplasmic antibody-associated vasculitis
ACEI: Angiotensin-converting enzyme inhibitors
ANA: Antinuclear antibodies
ARB: Angiotensin II receptor blocker
AZA: Azathioprine
BVAS: Birmingham Vasculitis Activity Score
cANCA: cytoplasmic antineutrophil cytoplasmic antibody
CI: 95% confidence intervals
CTX: Cyclophosphamide
eGFR: estimated glomerular filtration rate
EGPA: eosinophilic granulomatosis with polyangiitis
ELISA: Enzyme-linked immunosorbent assay
ENT: Ear nose and throat
ESR: Erythrocyte sedimentation rate
ESRD: End-stage renal disease
GPA: Granulomatosis with polyangiitis
HRs: Hazard ratios
IIF: Indirect immunofluorescence assay
IQR: Interquartile range
IV: Intravenous
MMF: Mycophenolate mofetil
MPA: Microscopic polyangiitis
MPO: Myeloperoxidase
pANCA: perinuclear antineutrophil cytoplasmic antibody
PCP: Pneumocystis jirovecii pneumonia
PMN: Polymorphonuclear leucocytes
PR3: Proteinase 3
RLV: Renal-limited vasculitis
SD: Standard deviation
